# Primary hyperparathyroidism in the geriatric population: A case report and mini literature review

**DOI:** 10.1002/ccr3.6313

**Published:** 2022-11-16

**Authors:** Priya Jaisinghani, Anupa Sharma, Xiangbang Wang

**Affiliations:** ^1^ Division of Endocrinology, Metabolism, and Nutrition, Department of Medicine New York University School of Medicine New Brunswick New Jersey USA; ^2^ Division of Endocrinology, Metabolism, and Nutrition, Department of Medicine Rutgers University‐Robert Wood Johnson Medical School New Brunswick New Jersey USA

**Keywords:** geriatric population, hypercalcemia, medical management, primary hyperparathyroidism

## Abstract

We report a case of a 93‐year‐old woman with PHPT secondary to a left inferior parathyroid adenoma. The patient met criteria to be a surgical candidate; however, literature about parathyroidectomy in the elderly was limited and controversial. The patient remained stable through medical management for the next 5 years.

## Abstract 2

Diagnosis and management of PHPT in the geriatric population remains challenging. Although parathyroidectomy is the definitive treatment in the general population, medical management can be strongly considered in the geriatric population with PHPT.

## 1 INTRODUCTION

Primary hyperparathyroidism (PHPT) is a hypercalcemic disorder involving the over activity of parathyroid glands resulting in excess secretion of parathyroid hormone (PTH). PHPT is one of the most common endocrine disorders and the most common cause of hypercalcemia with prevalence rates of about one to four per 1000 in the general population. The incidence of developing PHPT increases with age and is up to one per 100 in the elderly.^1^ According to the World Health Organization, the number of people aged 65 or older is projected to rise from an estimated 524 million in 2010 to nearly 1.5 billion in 2050.^2^ As the elderly population continues to grow, so should the focus on diseases that affect them, such as PHPT.

Most patients are asymptomatic and elevated calcium levels are only discovered on routine blood work. An elevated or inappropriately normal serum PTH value in the presence of true hypercalcemia is always abnormal and typically indicates the presence of PHPT.

Older asymptomatic patients with PHPT may have significant functional deficits that can affect their safety and quality of life compared with healthy age‐matched subjects. In long‐standing PHPT, there is a reduction in bone density that may lead to an increased risk of fractures. This is especially true in postmenopausal women who are already at risk for osteoporosis due to estrogen deficiency.^3^ Identifying and providing appropriate treatment for this at‐risk population is therefore crucial. The main focus of this review will be on diagnosis and management of PHPT in the elderly population (age 75 and older) through a case to illustrate the challenge of diagnosis and management of PHPT in geriatric population.

## 2 CASE REPORT

A 93‐year‐old woman with a past medical history of severe osteoporosis treated with IV infusion zoledronic acid/risedronate/vitD and calcium supplementation complicated by vertebral compression fractures, hypothyroidism, arthritis, gastroesophageal reflux disease, and obesity who was referred to endocrinology for evaluation and treatment of hypercalcemia. Patient's active medications at the time included levothyroxine 88 mcg qD, atenolol 25 mg qD, atorvastatin 20 mg qHS, aspirin 81 mg qD, cinacalcet 30 mg qD, risendronate 35 mg qweekly, vitamin D 400 IU qD, folic acid 800 mcg qD, ferrous gluconate 13.5 mg qDm, calcium citrate/magnesium 167/95 mg qD. Initial labs work showed a PTH of 165 pg/ml (10–65), creatinine 1.9 mg/dl (0.5–1.2), estimated glomerular filtration rate in non‐African American 26 (≥60), a serum calcium of 10.6 mg/dl (8.6–10.4), albumin 3.7 gm/dl (3.5–5.0), corrected calcium 11.2, 25‐hydroxyvitamin D level 29 ng/ml (20–50), parathyroid hormone related protein <2 pmol/L and 24 h urine calcium of 204. Most recent dual energy X‐ray absorptiometry **(**DXA) scan showed a T score of −2.6 of the right femoral neck which was a 4% interval decrease from the prior study. The patient was questionably symptomatic from the standpoint of hyperparathyroidism reporting symptoms of chronic constipation and significant arthritic pain. Otherwise, the patient was reporting no chest pain, shortness of breath, vocal changes, abdominal pain, chronic fatigue, difficulty sleeping, memory loss, poor concentration, headaches. On examination, the patient did not demonstrate any thyromegaly, thyroid nodules/densities, or lymphadenopathy. Parathyroid sestamibi scan was suggestive of left inferior parathyroid adenoma. Patient met the criteria for parathyroid surgery as her T‐score was −2.6. In 2002, the bone mineral density (BMD) of the lumbar spine (L1‐L4) was 0.900 gm/cm^2^, T‐score −2.3. The BMD of the right femoral neck was 0.665 gm/cm^2^, T –score 2.6. In 2003, the BMD of the lumbar spine was 0.858 gm/cm^2^, T‐score −2.6. The BMD of the right femoral neck was 0.606 gm/cm^2^, T score −3.1. Patient did not have an abdominal ultrasound completed during the initial visit as she did not have a history of nephrolithiasis; however, 1 year after presentation, she did have a renal artery duplex which did not note any hydronephrosis or abnormal shadowing calculus in the bilateral kidneys.

The patient was referred to a proper surgical specialist for removal of the adenoma. Upon review of results and examination, it was noted that although the patient could benefit from parathyroidectomy, at her age it was not absolutely essential. Following a long discussion with family and the primary care physician and given that the patient was quite active and healthy, it was decided not to proceed with parathyroidectomy. The patient's hyperparathyroidism was then medically managed. About 1 year later, the patient's Vitamin D continued to decrease to 21 ng/ml, and thus, high‐dose Vitamin D therapy 50,000 IU per week × 12 weeks was initiated which then corrected the Vitamin D deficiency. The patient was also on calcitriol Vitamin D3 400 IU 3 tablets daily. PTH increased and was elevated at 569 pg/ml with stable calcium of 10.4 mg/dl. The rise in PTH reflects both the patient's diagnosis of hyperparathyroidism and worsening chronic kidney disease (CKD). Patient was also evaluated by nephrology for evaluation of CKD Stage 3–4, during which it was noted that the patient had shown evidence of mild azotemia for years with her creatinine usually ranging from 1.7–2.0 mg/dl and never greater than 2.1 mg/dl. Biomarker N terminal telopeptide (NTX) was followed to evaluate for continued zoledronic acid treatment. NTX is a telopeptide of type I collagen for bone resorption, thus monitoring biochemical markers that reflect changes in the material property of bone indicate bone strength. Levels of bone markers can decrease rapidly with antiresorptive therapies and monitoring the levels after 3–6 months has been shown to be more strongly associated with fracture outcome than changes in BMD. Subsequently, the patient received one additional dose of zoledronic acid infusion and then it was decided that she did not need further infusions after stable NTX levels.^4^ The patient was recommended to start a weight bearing exercise program. Patient was also advised to increase fluid intake but was not able to follow through with this. Patient's calcium remained stable, and the patient did not develop any new symptoms throughout the rest of her course for the next 5 years. Patient was noted to have tertiary hyperparathyroidism with PTH 1008 pg/ml, creatinine 3.3 md/dl, vitamin D 30.4 ng/ml.

At age 101, patient was hospitalized post recent hip surgery for shortness of breath status post intubation for acute on chronic respiratory failure in the setting of undifferentiated shock. Patient deceased after cardiac arrest.

### 2.1 Discussion

This case report highlights that it is difficult to diagnose and manage PHPT in the elderly; however, the progression of primary hyperparathyroidism is slow suggesting that not all patients especially in the geriatric population require surgery. We will now review the literature about geriatric PHPT.

Prevalence of PHPT in geriatric population: PHPT is a common disease that increases in prevalence with advancing age. Prevalence peaks in women around 70–79 years of age (492 per 100,000) and in men over 80 years old (264 per 100,000). In a cohort study of 3014 Swedish men between the ages of 69 and 81 years, the prevalence of PHPT and impact of disturbed calcium homeostasis on BMD were evaluated. Siilin et al. found that the prevalence of PHPT in elderly men was 0.73%, which is lower than that described in the literature regarding women in the same age group. This study also reported significantly lower BMD at the total hip, femoral neck, and trochanter in the PHPT group compared with the controls.^3^, ^5^

#### 2.1.1 Clinical presentation of PHPT in elderly

Asymptomatic hypercalcemia or osteoporosis are the most common clinical presentation of PHPT at diagnosis in the elderly like our patient.

These asymptomatic patients may progress to displaying symptoms of the disease by 15‐years of prospective follow‐up. When patients become symptomatic, it is usually a combination of increased PTH and hypercalcemia. Anorexia, nausea, constipation, polydipsia, and polyuria are more closely linked to elevated serum calcium levels.^5^ The skeletal involvement in classical PHPT reflects the increase in osteoclastic bone resorption with fibrovascular marrow replacement, as well as increased osteoblastic activity.^6^ Morphometric vertebral fracture rates are increased in postmenopausal women with PHPT regardless of symptoms. Fracture risk is increased at nonvertebral (cortical) and vertebral (trabecular) sites. The finding of mild morphometric vertebral fractures by X‐ray, CT, MRI, or vertebral fracture assessment in an asymptomatic PHPT patient represents an indication for parathyroid surgery.^7^

Nephrolithiasis and nephrocalcinosis are the most common overt complications of PHPT. Kidney stones are reported in 10%–25% of patients with PHPT. Up to one‐third may have some degree of renal dysfunction, either a significant reduction in creatinine clearance or impaired concentrating or acidifying ability. It is not possible to predict, from biochemical measurements in blood or urine, which asymptomatic patients with hyperparathyroidism will go on to develop new stone disease. Stone‐formers are more likely to be hypercalciuric, but less than one‐third of hypercalciuric patients with hyperparathyroidism actually develop stones. Presence of nephrolithiasis or nephrocalcinosis on imaging even in an asymptomatic individual, is viewed as an indication for parathyroidectomy, to prevent further symptomatic stone disease.

The elderly are more likely to exhibit neurocognitive symptoms even at minimally elevated serum calcium levels including of mental focus, changes in cognition, depression, and overall reduced quality of life.^8^, ^9^ As many of these symptoms are nonspecific, it remains unclear if surgical intervention would be of clinical benefit.

Whether PHPT increases the risk for peptic ulcer disease and pancreatitis remains controversial. In addition, although PHPT is associated with a higher risk of hypertension, the hypertension has not been shown to be corrected post‐parathyroidectomy.^10^

In another study looking at the effects of elevated PTH on BMD, Blain et al. found that age is a strong predictor of a decline in BMD of the femoral neck in men and may be attributed to an age‐related increase in PTH.^11^

#### 2.1.2 Diagnosis of PHPT in the elderly patient

Primary hyperparathyroidism is often diagnosed in the sixth and seventh decade of life.^1^ As mentioned previously, majority of individuals diagnosed with PHPT are initially found to be hypercalcemic during an incidental screening test and most individuals are asymptomatic at presentation. Hypercalcemia with an elevated PTH level establishes the diagnosis. PTH levels rise with age and the normal range generally 10–65 pg/ml does not take this into account for the geriatric population.^12^, ^13^ Prior to confirming the diagnosis of PHPT, Vitamin D levels must be repleted as deficiency can cause secondary hyperparathyroidism, high bone turnover, bone loss, mineralization defects, and hip and other fractures.

A 24‐h urine calcium sample is not always required in the diagnosis of PHPT; however, can be useful in differentiating asymptomatic PHPT from familial hypocalciuric hypercalcemia (FHH). An elevated 24‐h urine calcium can also assess risk for renal complications such as nephrolithiasis in asymptomatic patients and serves as criteria for surgery in asymptomatic PHPT.

In addition, as seen in our case presentation, there are several other reasons for an elevated iPTH such as age, use of bisphosphonates, vitamin D deficiency, and renal insufficiency.

## 3 MANAGEMENT: SURGERY VS MEDICAL ALTERNATIVES FOR THE ELDERLY PHPT POPULATION

Surgical intervention is the only form of definitive treatment for PHPT regardless of age. When considering surgery, symptoms and complications of the disease are evaluated. However, the majority of elderly patient population is asymptomatic and less likely to develop complications of PHPT during the rest of their lifetime.^14^ In such cases, alternate therapeutic options other than surgery may be considered. Despite these guiding therapeutic principles, most of the literature on the treatment of PHPT is not age‐specific. There remains considerable variation and disagreement with regards to treatment of hyperparathyroidism as applied to the elderly population.

### 3.1 Surgery

Elderly patients can have minimally invasive parathyroidectomy performed as safely as their younger counterparts.^1^ This is due to the significant surgical advances being made. Minimally invasive parathyroidectomy results in a smaller incision, less cervical dissection, and decreased postoperative discomfort. Additionally improved imaging and localization studies as well as rapid intraoperative parathyroid hormone assay testing has become more accessible.

Even with these new developments, a progressive age‐related decline in parathyroid treatment rate has been observed that renders patients aged 70 years and older unlikely to have definitive treatment, irrespective of comorbidity and eligibility for surgery.^1^ In 2003, Kebebew and colleagues found that 22% of their PHPT patients older than 80 years of age who were referred for parathyroidectomy had a delay of more than a year before referral, with mean delay of 5 years. In this study, all patients were symptomatic by the time of referral.^1^

Criteria for surgery include significant hypercalcemia (>1 mg/dl above the upper limit of normal), marked hypercalciuria (>400 mg per day), low bone density, vertebral fracture found on imaging, unexplained renal insufficiency, or creatinine clearance of <60, presence of nephrolithiasis or nephrocalcinosis, or an episode of acute primary hyperparathyroidism.^4^, ^9^

According to the guidelines, the majority of elderly PHPT patients would qualify for surgery based on conditions such as osteoporosis or having a creatinine clearance of <60. Thus, much of the current literature advocates for more aggressive surgical therapy for the elderly. However, there is an increased risk for complications, prolonged operation time and increased length of stay in hospitals for patients over the age of 80 years.^15^ Politz et al. studied 150 patients over the age of 80 years who underwent parathyroidectomy and did not show evidence of symptomatic benefit after surgery.^16^ Morris et al supported a similar position in their literature review.^17^ At the extremes of age, particularly in the very elderly, the value of surgery at achieving cure is less straightforward than for the young. Our case supports that medical management may be an acceptable option for the elderly age group.

### 3.2 Medical management

The rate of progression of PHPT is slow and not all patients require surgery. In fact, a large number of elderly asymptomatic patients with PHPT can be monitored or even medically managed unless there is progression of disease. In a 10 year prospective study by Silverberg et al., PHPT patients who did not undergo parathyroid surgery remained stable during a decade of observation.^18^ Calcium concentrations, reductions in bone density, occurrences of fragility fracture, and renal endpoints are all followed closely.

For individuals who do not meet any of the surgical criteria or refuse surgery, skeletal monitoring is recommended by 3‐site BMD every 1–2 years.^19^ Patients with long‐standing PHPT have a reduction in BMD, particularly at predominantly cortical skeletal sites, such as the one‐third radius, with relative preservation of BMD at the lumbar spine. Some but not all studies have reported an increase in fracture risk with PHPT.^3^ The study of Rubin has shown stability for up to 8–9 years with nonsurgical observation but deterioration at the hip and distal 1/3 radius afterwards.^20^ In the elderly, no specific radiological manifestations are observed and skeletal X‐ray screening is not recommended. However, DXA scans of the hip, spine, and one‐third radius should be an integral part of the evaluation of individuals with PHPT.^21^

In a subset of symptomatic older individuals who meet surgical indications, surgery may not be a possibility due to coexisting medical complications.^11^ Frailty and comorbidity in the elderly population has made surgery a less desirable option. Elderly who are not able to undergo surgical management or wish not to undergo surgery should remain hydrated, avoid immobilization, and stop offending agents. In acute settings, severe hypercalcemia should be treated with rehydration, furosemide, and therapeutic agents such as bisphosphonates.

Literature in favor of medical management of the very old shows promise despite its smaller scope. Khan et al.^22^ and Marcocci et al.^7^, ^8^ reviewed the rationale and evidence behind medical management and expectant monitoring. They found that medical management can be effective, and that the patients' quality of life can be similar to those treated surgically. Under medical management, PTH and calcium can remain stable for 10 years. Thomas et al. conceded in a study of over 7000 patients that those over the age of 80 who underwent surgery had higher complication rates including death, length of stay, and operation time.^23^

Jacobs et al.^24^ described a case series of 4 patients with PHPT aged 79 to 87 years who were treated with medical management as a bridge to surgery or as sole therapy in poor surgical candidates or those refusing surgery. They employed saline hydration and pamidronate, and recommended cinacalcet as a second‐line therapy. Medical therapy was a successful bridge to surgical cure with parathyroidectomy for two patients after 2 weeks to 2 months of treatment.

#### 3.2.1 a. Bisphosphonates

Randomized controlled trials have shown that medical therapy, in particular treatment with a bisphosphonate, can be as effective as surgical intervention in increasing BMD and reducing bone turnover in PHPT patients.^25^ This provides an alternative option to surgery in patients with increased fracture risk.^26^

Bisphosphonates are powerful inhibitors of bone resorption and may be useful in improving low bone mass in patients who have not undergone parathyroidectomy. Iv zoledronic acid has FDA approval for osteoporosis and also decreases serum calcium by 1 mg/dl at 3 months. More potent bisphosphonates such as alendronate or risedronate are used particularly in the elderly with comorbidities that put them at increased surgical risk.^26^ Alendronate in particular has been extensively studied in patients with PHPT.

Four different studies demonstrated alendronate use in mild PHPT over one to two years, resulting in an increase in bone density at the hip and lumbar spine.^15^, ^27^, ^28^, ^29^ Chow et al. showed a decline in bone turnover markers in their particular study.^28^ However, serum calcium and PTH levels remain relatively unchanged.^30^ One study in particular by Rossini et al. evaluated postmenopausal women aged 67–81, resulting in statistically significant increases in BMD after 2 years of alendronate therapy.^31^ Data on use of bisphosphonates other than Alendronate in PHPT is limited. Currently, there is no fracture data regarding bisphosphonate therapy in PHPT patients.

#### 3.2.2 b. Calcimimetics

Calcimimetics are a class of medications that alter the function of calcium‐sensing receptors by making them more sensitive to extracellular calcium, and therefore, reducing calcium and PTH levels in the serum.^32^ In patients with refractory PHPT after parathyroidectomy or with contraindications to surgery, calcimimetics such as Cinacalcet can be an effective form of therapy in normalizing serum calcium levels.

Peacock et al. reported a placebo‐controlled randomized clinical trial demonstrating the ability of cinacalcet to normalize serum calcium levels in patients with PHPT over a 1‐year period.^33^ An open‐label extension study of this trial was conducted, resulting in reduction of serum calcium levels compared with baseline and maintained normocalcemia with reduced serum PTH levels over a 5‐year period.^34^ In an open‐label, single‐arm study by Marcocci et al. the dose of cinacalcet was titrated upwards until efficacy was achieved, and almost 90% of patients with severe PHPT resulted in a 1 mg/dl or greater reduction in serum calcium levels.^35^

A meta‐analysis by Leere et al. consisting of 19 studies measured plasma calcium response to Cinacalcet vs bisphosphonates over a duration ranging from 15 days to 44 months. Cinacalcet proved to be effective in lowering plasma calcium levels and did not lose its calcium‐lowering capability over time in contrast to bisphosphonates which have a short‐lived (<6 months) effect on plasma calcium. Cinacalcet also had some effect on lowering serum PTH and urinary calcium excretion, the latter possibly having an attenuating effect on renal stone disease. Unlike with bisphosphonate use, there was no change in bone turnover and there was no improvement in BMD.^36^

There is very limited data on long‐term effects of Cinacalcet due to the short period of time it has been available. Calcimimetics are generally well tolerated14; however, the most common adverse effects include nausea, vomiting, and paresthesias.^35^ There is no improvement seen in BMD and can actually increase markers of bone turnover, and therefore, is not suggested for fracture risk management in PHPT.^33^, ^34^

#### 3.2.3 c. Estrogen and estrogen modulators in the elderly

Many patients with PHPT are postmenopausal women at risk for osteoporosis due to estrogen deficiency.^3^ Estrogen therapy reduces PTH‐mediated bone resorption, reduces urinary calcium excretion, and increases bone mineral density. However, it is not widely accepted as a treatment option in older women because of its safety profile (increased risk for contrary heart disease, breast cancer, blood clots, and stroke) as well as its minimal effect on serum calcium and PTH. Raloxifene is selective estrogen receptor modulator (SERM) that has estrogenic actions on bone and was the first SERM to be approved for management of postmenopausal osteoporosis.^37^

McDermott et al. reported a cross‐sectional study of 59 asymptomatic women with PHPT it was found that those treated with HRT had higher BMD results than those not on HRT in both the PHPT and the control group.^38^ In osteoporotic postmenopausal women, a study by Sanad et al. compared alendronate and raloxifene, either alone or in combination, showing significant increase in BMD of the lumbar spine, femoral neck and total hip and reduced markers of bone turnover in all treatment groups.^39^ Raloxfiene also showed a significant decrease in serum calcium levels suggesting that it could be used for the short‐term control of calcium levels in patients awaiting surgery.^40^, ^41^, ^42^, ^43^

## 4 CONCLUSION

The approach to diagnosis and treatment of PHPT in the elderly should be individualized and tailored to each patient but medical management can be strongly considered in many cases. Although surgery is the definitive treatment of PHPT in the geriatric population, asymptomatic patients can be monitored and medically managed. In addition, frailty and comorbidities in the elderly population may make them less suitable candidates for surgical intervention due to increased surgical risk. Finally, medical management includes supportive measures such hydration and mobilization, and follow‐up of calcium concentrations, bone density, and renal endpoints. Further studies in this area will provide valuable information regarding the advantages of medical management of PHPT in the geriatric population.

## AUTHOR CONTRIBUTIONS

Dr. Priya Jaisinghani wrote the majority of the manuscript while all co‐authors critically reviewed and revised the manuscript.

## FUNDING INFORMATION

No funding was required for this review.

## CONFLICT OF INTEREST

The authors have no conflicts of interests to disclose.

## ETHICAL APPROVAL

Ethical approval was not required for this review.

## CONSENT

Written informed consent was obtained from the patient to publish this report in accordance with the journal's patient consent policy.

## Data Availability

The data that support the findings of this study are available from the corresponding author upon reasonable request.
